# Photosynthesis Is Widely Distributed among Proteobacteria as Demonstrated by the Phylogeny of PufLM Reaction Center Proteins

**DOI:** 10.3389/fmicb.2017.02679

**Published:** 2018-01-23

**Authors:** Johannes F. Imhoff, Tanja Rahn, Sven Künzel, Sven C. Neulinger

**Affiliations:** ^1^Research Unit Marine Microbiology, GEOMAR Helmholtz Centre for Ocean Research, Kiel, Germany; ^2^Max Planck Institute for Evolutionary Biology, Plön, Germany; ^3^omics2view.consulting GbR, Kiel, Germany

**Keywords:** photosynthetic reaction center proteins, Proteobacteria, phototrophic purple bacteria, photosystem II, phylogeny, anoxygenic photosynthesis, species recognition, bacteriochlorophyll *b*

## Abstract

Two different photosystems for performing bacteriochlorophyll-mediated photosynthetic energy conversion are employed in different bacterial phyla. Those bacteria employing a photosystem II type of photosynthetic apparatus include the phototrophic purple bacteria (Proteobacteria), *Gemmatimonas* and *Chloroflexus* with their photosynthetic relatives. The proteins of the photosynthetic reaction center PufL and PufM are essential components and are common to all bacteria with a type-II photosynthetic apparatus, including the anaerobic as well as the aerobic phototrophic Proteobacteria. Therefore, PufL and PufM proteins and their genes are perfect tools to evaluate the phylogeny of the photosynthetic apparatus and to study the diversity of the bacteria employing this photosystem in nature. Almost complete *pufLM* gene sequences and the derived protein sequences from 152 type strains and 45 additional strains of phototrophic Proteobacteria employing photosystem II were compared. The results give interesting and comprehensive insights into the phylogeny of the photosynthetic apparatus and clearly define Chromatiales, Rhodobacterales, Sphingomonadales as major groups distinct from other Alphaproteobacteria, from Betaproteobacteria and from Caulobacterales (*Brevundimonas subvibrioides*). A special relationship exists between the PufLM sequences of those bacteria employing bacteriochlorophyll *b* instead of bacteriochlorophyll *a*. A clear phylogenetic association of aerobic phototrophic purple bacteria to anaerobic purple bacteria according to their PufLM sequences is demonstrated indicating multiple evolutionary lines from anaerobic to aerobic phototrophic purple bacteria. The impact of pufLM gene sequences for studies on the environmental diversity of phototrophic bacteria is discussed and the possibility of their identification on the species level in environmental samples is pointed out.

## Introduction

Bacteriochlorophyll-mediated anoxygenic photosynthesis is widely distributed among eubacteria and found in the Chlorobi, Firmicutes (Heliobacteria), and Chloracidobacteria employing a type-I photosystem as well as in Proteobacteria, Chloroflexi, and Gemmatimonadetes employing a type-II photosystem (Zhang et al., 2003; Madigan et al., 2017; Zeng and Koblizek, 2017). Most likely, the two photosystems have evolved in different ancestors of our present day phototrophic bacteria prior to their combination in the primordial cyanobacteria, which made possible for the first time the use of water as electron source for photosynthesis and the evolution of oxygen. As the anoxygenic photosynthesis presumably has been established about 3.2–3.5 billion years ago (Blankenship, 2010), it can be assumed that the two photosystems have evolved more than 3 billion years before present and further diversified since then.

In this study, we focus on the phototrophic Proteobacteria, also known as the phototrophic purple bacteria. The phototrophic Proteobacteria, traditionally represented by the anaerobic anoxygenic phototrophic purple bacteria (with the distinction of purple sulfur and purple non-sulfur bacteria), are living and performing photosynthesis under anoxic conditions (Pfennig, 1977; Drews and Imhoff, 1991). In addition, a large group of Proteobacteria performing anoxygenic photosynthesis under oxic conditions is abundant and of ecological importance in aquatic habitats, in particular in ocean waters (Imhoff and Hiraishi, 2005; Yurkov, 2006; Yurkov and Hughes, 2017). The first representatives thereof have been isolated more than 40 years ago (Shiba et al., 1979; Shiba and Simidu, 1982; Shiba, 1991). The ability to perform photosynthesis is deeply rooted within the Proteobacteria and found in both anaerobic as well as aerobic phototrophic purple bacteria. Representatives of the anaerobic phototrophic purple bacteria form distinct lineages in the Alphaproteobacteria (the orders Rhodospirillales, Rhizobiales, and Rhodobacterales), in the Betaproteobacteria (the orders Burkholderiales and Rhodocyclales) and in the Gammaproteobacteria (the order Chromatiales). The aerobic phototrophic purple bacteria add to the phylogenetic diversity with representatives in most of the aforementioned orders and in addition with distinct groups in the Cellvibrionales, Sphingomonadales, and Caulobacterales, that lack known anaerobic phototrophic representatives.

As an essential component of photosystem II, the core of the photosynthetic reaction center, is formed by two membrane spanning proteins (PufL and PufM) which are binding bacteriochlorophyll molecules. The conserved nature of these reaction center proteins and their common presence in all phototrophic bacteria employing photosystem II make these genes perfect tools for the analysis of the phylogenetic relationship of these bacteria. In addition, the *pufLM* genes are excellent tools to specifically trace the environmental diversity of phototrophic bacteria employing photosystem II, in particular because the 16S rRNA gene is not suited to analyze the environmental diversity of phototrophic bacteria (Tank et al., 2009, 2011; Thiel et al., 2010; Imhoff, 2017). Though in a few environmental case studies almost complete *pufLM* sequences have been successfully applied to study the diversity of purple sulfur bacteria (Gammaproteobacteria) and identify species and their relatives (Tank et al., 2009, 2011; Thiel et al., 2010), a comprehensive data base of type and reference strains from all groups of phototrophic purple Proteobacteria is lacking. Therefore, almost complete *pufLM* gene sequences and the derived protein sequences from 152 type strains and 45 additional strains, which are included as replacements for unavailable types or are strains of interest for other reasons, were compared in the present study. The results give interesting and comprehensive insights first of all into the phylogeny of the photosynthetic apparatus and secondly strongly support environmental studies on the diversity of phototrophic purple bacteria enabling their identification in environmental samples on a species-specific level.

## Materials and Methods

### Sequence Retrieval

Amino acid sequences from *pufL* and *pufM* genes were retrieved from the NCBI database NCBI and from sequences of own ongoing genome projects (all sequences are shown in **Supplementary Table S1**).

### Sequences from Ongoing Studies

Additional sequences of *pufL* and *pufM* genes were obtained in the present study from an ongoing genome project. Genome sequences were annotated using the Rapid Annotations using Subsystems Technology (RAST, Aziz et al., 2008; Overbeek et al., 2014). Sequences of *pufL* and *pufM* were retrieved from the annotated genomes and deposited in the EMBL database under the accession numbers MF563614–MF563648, MG010630–MG010637, and MG596305–MG596307) (*pufL*) and MF563649–MF563683, MG010638–MG010645, and MG596308–MG596310 (*pufM*) as shown in **Supplementary Table S1**.

### Phylogenetic Analyses

Multiple sequence alignments (MSAs) were produced with MAFFT v7.305b (Katoh et al., 2002; Katoh and Standley, 2013) from all PufL and PufM sequences and were visually inspected for consistency. MAFFT was run with parameters ‘–globalpair –maxiterate 1000.’ Sequences in both PufL and PufM MSAs were trimmed to the same length. Alignment positions with >10% gaps were removed resulting in a final alignment lengths of 214 aa (PufL) and 180 aa (PufM), respectively. PufL and PufM MSAs were concatenated to form a single PufLM MSA. A maximum likelihood (ML) phylogenetic tree was calculated from the PufLM MSA with the program IQ-TREE v1.4.4 (Nguyen et al., 2014), using separate substitution models for PufL and PufM alignment parts (so-called partition models, Chernomor et al., 2016). Optimal amino acid substitution models were determined to be LG+R7 (PufL part) and LG+F+R5 (PufM part). The aligned sequences are found in **Supplementary Materials**.

Ultrafast bootstrap approximation (UFBoot) (Minh et al., 2013) was used to provide branch support values with 1,000 replicates based on the same substitution models as the original ML tree. Branch support values were assigned onto the original ML tree as the number of times each branch in the original tree occurred in the set of bootstrap replicates (IQ-TREE option ‘-sup’).

The calculated phylogenetic tree was midpoint-rooted with the R package phangorn v2.0.4 (Schliep, 2011). For tree visualization iTOL v3.4.3 was used (Letunic and Bork, 2016). Bootstrap values within a range of 95–100% were represented as filled circles of varying size. For better visualization the circular tree was rearranged without altering its topology and divided into three subset trees.

## Results and Discussion

### Strain and Sequence Selection

For consideration in this study primarily type strains of phototrophic Proteobacteria were selected. Based on this consideration, a total of 197 PufLM sequences have been selected of which 152 were from type strains of validly described species and 19 from additional strains of some of these species, such as *Halorhodospira halophila* (three strains), *Allochromatium vinosum* MT86, *Thiocystis violacea* DSM 208, *Rhodospirillum rubrum* (three strains) *Rhodopseudomonas palustris* (three strains), *Rhodovulum sulfidophilum* DSM 2351, *Rhodocyclus tenuis* IM 230, *Afifella marina* (two strains) and *Rubrivivax gelatinosus* (four strains). In a few cases type strains (or sequences thereof) were not available, have been lost or neotypes have not been described [*Thiococcus pfennigii* (four strains), *Chromatium weissei, Rhodobacter sphaeroides, Phaeospirillum fulvum, Rhodocista centenaria*] or species have not been validly described (“*Thiocapsa bogorovii*” BBS, “*Ectothiorhodospira imhoffii*,” “*Rhodobacter weaveri*” TJ-12). In order to establish their relationship to the reference strains, sequences of the following strains have been included into the study: (i) *Rhodoferax fermentans* the data base entry of which lacks a strain assignment, (ii) some unidentified strains with unclear affiliations to known species (*Bradyrhizobium* sp. BTAi1, *Thiohalocapsa* sp. ML1, *Thiorhodovibrio* sp. 970, *Ectothiorhodospira* sp. PHS-1, *Ectothiorhodospira* sp. BSL-9) and (iii) five strains related to *Chloroflexus/Roseiflexus*. Though the data base entries available for three *Acidiphilium* species (*Acp. rubrum, Acp. cryptum, Acp. organovorum*) were without strain assignment, they were also included because alternative sequences were not available. In all other instances sequences without given identification of species and/or strain numbers were excluded from this study. The sequences together with the species and strain designation and their systematic treatment are compiled in the **Supplementary Table S1**.

### The Phylogeny of PufLM

**FIGURE 1:**
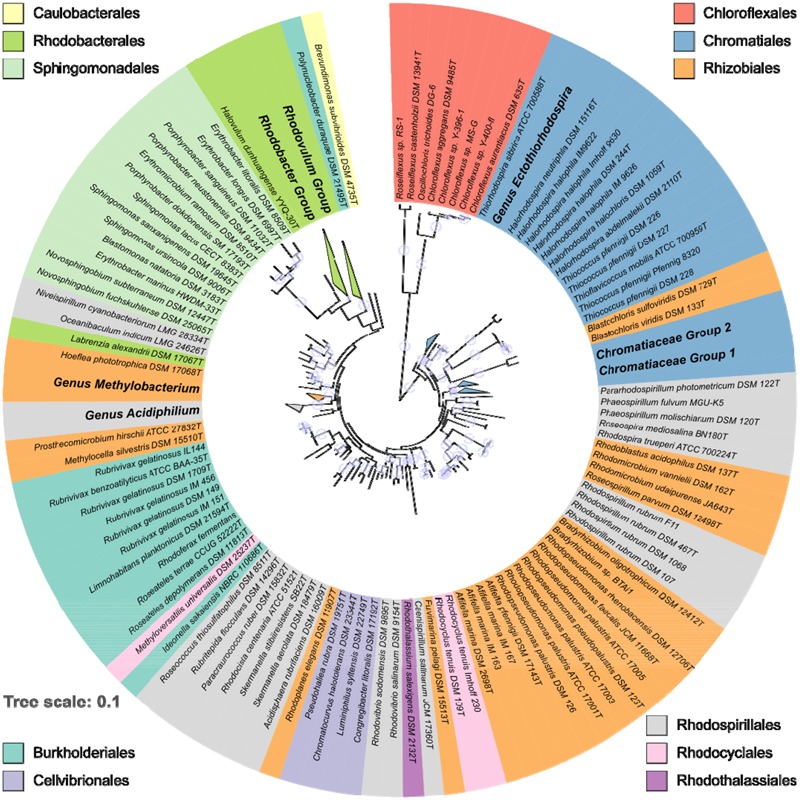
Overview on the phylogeny of PufLM sequences from 197 aerobic and anaerobic phototrophic bacteria (152 type strains). For convenience, major homogenous groups have been condensed in this tree. Details thereof are shown in **Figures 2A–C**. The color code identifies the belonging to different orders. Bootstrap values within a range of 95% - 100% are represented with colored circles of varying size.

**FIGURE 2:**
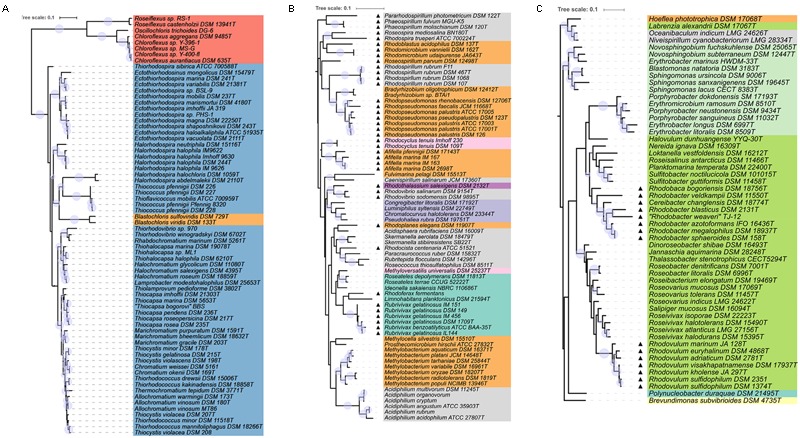
Detailed view of the phylogenetic tree of PufLM sequences. The color code for different orders is the same as used for **Figure 1**. Bootstrap values within a range of 95% - 100% are represented with colored circles of varying size. **(A)** Shows the part of Gammaproteobacteria together with Chloroflexi and the species containing bacteriochlorophyll *b*. **(B,C)** Show anaerobic anoxygenic phototrophic Alpha- and Betaproteobacteria marked with triangles and the aerobic anoxygenic phototrophic bacteria of all groups of Proteobacteria.

The distribution and phylogenetic relationship of the *pufLM* genes among the Proteobacteria reveals that the ability to synthesize a reaction center and to perform photosynthesis is deeply rooted among these bacteria. Representatives of the anaerobic phototrophic purple bacteria form distinct lineages in Alpha-, Beta-, and Gammaproteobacteria: in the orders Rhodospirillales, Rhizobiales, and Rhodobacterales, in the Burkholderiales, Rhodocyclales, Rhodothalassiales, and Chromatiales (**Figure 1**). As revealed by PufLM sequences, the aerobic phototrophic purple bacteria add to the phylogenetic diversity with groups containing exclusively aerobic phototrophic bacteria as represented by Cellvibrionales, Sphingomonadales, and Caulobacterales (see **Figures 1, 2**). It is quite remarkable to see that most of the major systematic groups form consistent branches within the PufLM tree. These include the Chromatiales, the Rhodobacterales (with the exception of *Labrenzia alexandrii*), the Cellvibrionales, the Sphingomonadales and the Burkholderiales (with an exceptional position of *Polynucleobacter duraquae*). The Rhodothalassiales are represented by a single species, *Rhodothalassium salexigens*, which has a PufLM sequence well distinct from other Alphaproteobacteria with some distant relationship to the two *Rhodovibrio* species. Also the Caulobacterales are represented by *Brevundimonas subvibrioides* as a single species, and clearly fall apart from all other sequences in the tree (**Figures 1, 2C**). The Rhodocyclales are represented by *Methyloversatilis universalis*, which associates with the Burkholderiales and by *Rhodocyclus tenuis*, which (together with *Rhodocyclus purpureus*, data not shown) holds a separate position within the tree. The situation is more complex within the orders Rhodospirillales and Rhizobiales. Though at the genus level clearly distinct lineages are formed within these orders too, they have no well-defined roots within the tree (see below). A special situation applies to the sequences from bacteria employing bacteriochlorophyll *b* in the reaction center as discussed below.

Sequences of PufLM from representative Chloroflexi, that were included into the phylogenetic considerations, are by far the most distant to all PufLM sequences from other bacteria employing photosystem II and forming two distantly related branches with representatives of *Roseiflexus* in one and representatives of *Chloroflexus* together with *Oscillochloris* in a second branch (**Figures 1, 2A**). It is quite obvious, that the phylogenetic distance of Chloroflexi to the purple bacteria is tremendous and it is tempting to conclude that these bacteria and their photosystem have the most ancient roots and evolved much earlier than the system in purple bacteria (Gaisin et al., 2016).

#### Chromatiales/Ectothiorhodospiraceae and Chromatiaceae

With the exception of those species which have bacteriochlorophyll *b* as major pigment in the reaction center, the phylogenetic relationship of PufLM sequences is largely congruent with the 16S rRNA gene phylogeny of the two phylogenetically well separated families of the phototrophic Gammaproteobacteria, the Ectothiorhodospiraceae and Chromatiaceae (Imhoff, 2017). Different lineages in the Ectothiorhodospiraceae are represented by the genera *Ectothiorhodospira* (*Ectothiorhodosinus* associated to it) and *Halorhodospira*. *Thiorhodospira sibirica* forms a separate branch only distantly related to the *Ectothiorhodospira* species, which is in accordance to its relationship seen with 16S rRNA gene sequence (Imhoff, 2017).

Smaller phylogenetic distances but a robust formation of clusters also is characteristic for the Chromatiaceae. A cluster of halophilic species contains groups of *Thiorhodovibrio* and *Rhabdochromatium* species on one hand and those of *Thiohalocapsa, Halochromatium*, and *Lamprobacter* on the other hand. Within a separate cluster, groups are represented by the genera (i) *Thiocapsa* and *Thiolamprovum*, (ii) *Marichromatium*, (iii) *Thiocystis* (*Tcs. violascens, Tcs. minor, Tcs. gelatinosa*, but not including the type species *Tcs. violacea*) and *Chromatium*, (iv) *Thiorhodococcus* and *Thiocystis violacea* (the type species) and (v) *Thermochromatium* and *Allochromatium* species. It must be emphasized that species of *Thiocystis* fall into two subgroups and species of *Thiorhodococcus* into different subgroups of group (iv) (**Figures 1, 2A**). The groupings recognized by PufLM sequences as outlined before are almost identical to those found according to the 16S rRNA gene sequences (Imhoff, 2017). The heterogeneity of *Thiorhodococcus* and *Thiocystis* species, e.g., is documented by both *pufLM* sequences and 16S rDNA phylogeny as well (Imhoff, 2017). Accordingly, *Thiorhodococcus drewsii* and *Thiorhodococcus kakinadensis* (together with *Trc. fuscus*) fall apart from other species (*Trc. minor* and *Trc. mannitoliphagus*) of this genus. Similarly, *Thiocystis violacea* (DSM 207^T^), the type species of this genus, stands apart from other *Thiocystis* species that group together with the *Chromatium* species.

#### Rhodobacterales/Rhodobacteraceae

Like the Chromatiales, the large number of phototrophic species known of the Rhodobacterales form a coherent group and the PufLM phylogeny of the Rhodobacterales is mostly congruent with that of the 16S rRNA gene. Both anaerobic and aerobic types of phototrophic bacteria are found in this order. *Rhodovulum, Rhodobacter*, and *Rhodobaca* are representatives of the anaerobic phototrophic life style, though most species of these genera are metabolically highly versatile and can proliferate using phototrophic as well as chemotrophic ways of energy generation (Imhoff, 2005). Two large clusters are formed including *Rhodovulum* and *Rhodobacter* species, respectively. The genus *Rhodovulum* forms a well distinguished subgroup as do the *Roseivivax* and *Roseovarius* species, with *Salipiger mucosus* specifically associated to *Roseovarius indicus*, but apart from the two other *Roseovarius* species that distantly associate with *Roseibacterium elongatum*. Additional subgroups are represented by species of (i) *Roseobacter* and (ii) *Dinoroseobacter, Jannaschia*, and *Thalassobacter*. Within the *Rhodobacter* cluster subgroups are represented by species of *Rhodobacter* associated to *Rhodobaca, Sulfitobacter* associated to *Planktomarina*, and a group of species not closely related to each other including those of *Roseisalinus, Loktanella, Halovolum*, and *Nereida*. Sequences of *Labrenzia alexandrii* are not closely related to any of these groups of species of the Rhodobacteraceae (see below) and this distinct position is supported by 16S rRNA gene analyses (data not shown).

#### Sphingomonadales/Sphingomonadaceae and Erythrobacteraceae

Another major and clearly distinct group of sequences is represented by the Sphingomonadales, which at present exclusively include aerobic phototrophic bacteria. Two distinct clusters of sequences represent the two families Sphingomonadaceae (*Sphingomonas, Novosphingobium, Blastomonas*) and Erythrobacteraceae (*Erythrobacter, Porphyrobacter, Erythromicrobium*). As an outlier, the sequences from *Erythrobacter marinus* HWDEM-33 do not cluster with the other *Erythrobacter* species and with the family Erythrobacteraceae, but with the *Novosphingobium* species of the Sphingomonadaceae. This is in accord with other phylogenetic analyses using concatenated amino acid sequences of 27 universally conserved proteins and 16S rRNA gene sequences demonstrating that *Erythrobacter marinus* is associated with Sphingomonadales and is phylogenetically distant to the established *Erythrobacter* species around *Erythrobacter longus*, the type species of the genus (Zheng et al., 2016). This actually points to a misclassification of *Erythrobacter marinus* and related species rather than to other explanations.

#### Cellvibrionales/Halieaceae

A group of aerobic phototrophic Gammaproteobacteria is represented by four species of Halieaceae in the Cellvibrionales order. The group includes *Chromatocurvus halotolerans, Pseudohaliea rubra, Luminiphilus syltensis*, and *Congregibacter litoralis* and is poorly rooted in the phylogenetic PufLM tree. In all PufLM phylogenetic trees calculated it forms a consistent group, the roots of which appear closer to those of the Ectothiorhodospiraceae and the *Thiococcus/Thioflavicoccus* group than to other phototrophic bacteria. The distinct but stable relationship of the sequences from these four species to each other and the distant association to other groups of the tree are characteristics of an ancient lineage that may have separated early from other lines and shares a basic set of common amino acids to all of the groups. It is not indicative of lateral gene transfer from any of the presently known phototrophic bacteria or more recent ancestors thereof.

#### Rhodospirillales and Rhizobiales

Most of the bacteria of these two orders form robust groups of PufLM sequences on the level of the genera, though both orders appear phylogenetically highly diverse. Clearly visible groups are recognized of the genera *Afifella, Rhodopseudomonas, Bradyrhizobium, Methylobacterium, Rhodospirillum, Rhodovibrio*, and *Acidiphilium*. Closely related genera are represented by *Bradyrhizobium* and *Rhodopseudomonas* species and by *Rhodoferax* and *Rubrivivax* species, respectively. The high diversity and our limited knowledge thereof are also depicted in the large number of genera that are represented by a few or even single species only, respectively, by the availability of only single PufLM sequences. Examples of such genera include *Roseospira, Rhodoplanes, Rhodospira, Roseospirillum, Pararhodospirillum, Fulvimarina, Caenispirillum, Rhodothalassium, Acidisphaera, Rhodocista, Paracraurococcus, Rubritepida, Roseococcus, Methylocella*, and *Prosthecomicrobium* (more than half of the genera of these two orders). Bacteria of these orders apparently represent an extremely diverse assemblage of bacteria and may have very ancient roots that cannot be clearly resolved based on the available PufLM sequence information (**Figures 1, 2**). Quite a number of the species from these genera, e.g., *Roseospirillum parvum* and *Rhodothalassium salexigens*, also form separate and deeply rooting lines in the phylogenetic tree of 16S rRNA gene sequences and have to be considered as species of distant relationship to other phototrophic Alphaproteobacteria (Imhoff, 2017).

#### The Hoeflea/Oceanibaculum – Group

A special group of PufLM sequences, that forms a stable and robust/coherent cluster, is found in four species that appear to be systematically unrelated: in *Hoeflea phototrophica* (Rhizobiales, Phylobacteriaceae), *Labrenzia alexandrii* (Rhodobacterales, Rhodobacteraceae), and *Oceanibaculum indicum* and *Niveispirillum cyanobacteriorum* (both Rhodospirillales, Rhodospirillaceae). At first glance this group appears as a misclassified array of species or calls for lateral gene transfer as an explanation. Indeed, the phylogeny of 16S rRNA gene sequences reveals that *Hoeflea phototrophica* and *Labrenzia alexandrii* (type strains) do not fit into any other group among the phototrophic Alphaproteobacteria, but form a deeply rooted separate cluster (data not shown). *Hoeflea phototrophica* is the only representative of the Phyllobacteraceae (Rhizobiales) and *Labrenzia alexandrii* is only distantly related to other Rhodobacteraceae. Also 16S rRNA gene sequences of *Oceanibaculum indicum* are only poorly associated to other Rhodospirillaceae such as *Skermanella* and *Rhodocista* species. This actually indicates that their classification is presently not unambiguous, which fits with their extraordinary position in the PufLM tree. Any discussion on lateral gene transfer for their separate position in the PufLM tree thus would be premature.

#### Burkholderiales and Rhodocyclales (Betaproteobacteria)

Based on the few sequences available, the genera of the phototrophic Betaproteobacteria form robust clusters, but their rooting within the tree is unclear. The Burkholderiales with species of *Rhodoferax* and *Limnohabitans* (Comamonadaceae) and of *Rubrivivax, Ideonella*, and *Roseateles* (unclass. Burkholderiales) form a larger cluster, including *Methyloversatilis universalis*, one of the *Rhodocyclaceae* species. *Polynucleobacter duraque* (Burkholderiaceae) is an outstanding single species with its PufLM sequence and only distantly related to other Betaproteobacteria. Also *Rhodocyclus* species (including *Rcy. purpureus*, data not shown) form a cluster apart from other phototrophic Betaproteobacteria. The majority of the known genera (*Methyloversatilis, Rhodoferax, Limnohabitans, Ideonella*, and *Polynucleobacter*) are represented by PufLM sequences from a single species. Therefore, the phylogenetic considerations within this group suffer from the low number of known representatives, respectively, PufLM sequences thereof. The phylogenetic distance between the known representatives, however, is suggestive of the existence of a much larger group not yet known or extinct over the past millennia.

#### Bacteria with Bacteriochlorophyll *b*

Of particular interest is the group of bacteria that have bacteriochlorophyll *b*, but not bacteriochlorophyll *a* as major photosynthetic reaction center pigment. Species of this diverse group of species from which PufLM sequences are known include *Halorhodospira halochloris* and *Halorhodospira abdelmalekii* as representatives of the Ectothiorhodospiraceae, *Blastochloris viridis* and *Blastochloris sulfoviridis* as representatives of the Rhizobiaceae, *Rhodospira trueperi* as representative of the Rhodospirillaceae and *Thiococcus pfennigii* and *Thioflavicoccus mobilis* as representatives of the Chromatiaceae. With the exception of *Rhodospira trueperi*, they show significant differences in the PufLM sequences compared to their respective relatives containing bacteriochlorophyll *a*. In the phylogenetic PufLM trees (**Figures 1, 2A**) they form a cluster of distantly related sequences apart from the other photosynthetic bacteria. Three clearly separate groups include species of *Halorhodospira* species (Gammaproteobacteria/Ectothiorhodospiraceae), of *Blastochloris* species (Alphaproteobacteria/Rhizobiales/Hypho microbiaceae) and of *Thiococcus* and *Thioflavicoccus* (most likely including those of *Thioalkalicoccus* as well (no data available), Gammaproteobacteria/Chromatiaceae).

**FIGURE 3:**
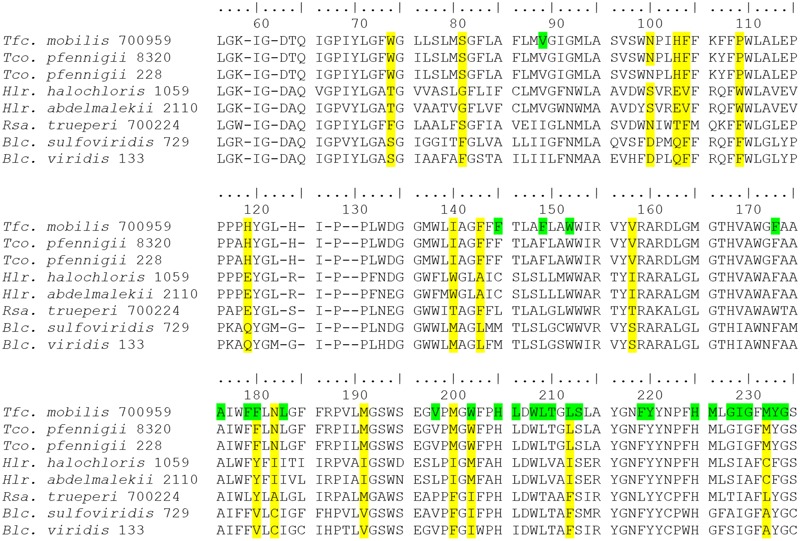
Alignment of PufM sequences (position 55–235) from bacteriochlorophyll-*b*-containing phototrophic bacteria. Important differentiating amino acids of the three groups are highlighted in yellow, bacteriochlorophyll-binding sites in green (only top line).

On close inspection of the sequences it becomes obvious that the three groups of bacteriochlorophyll-*b*-containing species have replacements of amino acids compared to their bacteriochlorophyll-*a*-containing relatives in quite a number of positions including conserved binding sites of the bacteriochlorophyll (Marchler-Bauer et al., 2017). Quite characteristically, amino acid replacements found at particular sequence positions are different in the three groups of species, thus allowing the recognition of the groups based on their unique amino acid sequence (**Figure 3**). From the primary data of the amino acid sequences it is concluded, that the three groups of bacteria employing bchl *b* in the reaction center have evolved independently and represent separate phylogenetic lineages. As mentioned before, *Rhodospira trueperi* is an exception among the strains employing bacteriochlorophyll *b* as its PufM sequence does not show characteristic multiple exchanges of amino acids in significant positions if compared to relatives of the Rhodospirillaceae employing bacteriochlorophyll *a*. Most of the 33 highly conserved binding sites for the bacteriochlorophyll in PufM of this species are shared with other Rhodospirillaceae, in particular with *Roseospira mediosalina* and *Roseospirillum parvum*. Despite of a number of highly conserved positions of the PufM protein, the groups of bacteria with bacteriochlorophyll *b* lack larger stretches of common amino acids in the sequence, but rather have individual replacements different from group to group. Therefore, we assume that the biosynthesis of bacteriochlorophyll *b* may have been acquired several times and initiated structural adaptations of the reaction center proteins independently in the groups of species mentioned before. Furthermore, from the sequence information available and according to the discussion before, we clearly exclude the possibility of lateral gene transfer as a reason for gaining the functional properties coming along with the biosynthesis of bchl *b*, but consider the observed sequence relationships as a sign of convergent evolution.

### Phylogenetic Aspects of Aerobic Anoxygenic Photosynthesis

During the past decades, it has been realized that a large group of aerobic phototrophic purple bacteria exists that play an important role in aquatic habitats and in ocean waters (Shiba et al., 1979; Shiba and Simidu, 1982; Shiba, 1991; Imhoff and Hiraishi, 2005; Yurkov, 2006; Yurkov and Hughes, 2017). In particular the information from *pufLM* genes has supported the finding of new forms of aerobic phototrophic purple bacteria in recent years. Some of these bacteria performing photosynthesis form new phylogenetic lines as represented by the Sphingomonadales and the Caulobacterales with *Brevundimonas subvibrioides* as the only known photosynthetic representative so far. In addition, a large number of aerobic representatives related to major phylogenetic lines of known anaerobic phototrophic purple bacteria in Alpha-, Beta-, and Gammaproteobacteria were identified. Altogether, the aerobic anoxygenic phototrophic bacteria have representatives in all groups of Proteobacteria and are phylogenetically more diverse even than their classical anaerobic counterparts (**Figures 1, 2**).

If we consider the evolution of photosystems I and II as preconditions for the combination of both systems to perform oxygenic photosynthesis in the cyanobacteria, strictly anaerobic ancestors of anoxygenic phototrophic bacteria should have evolved prior to the establishment of oxygenic photosynthesis and represent the first phototrophic forms of life on earth. The aerobic forms of anoxygenic phototrophic bacteria should be late offsprings of the anaerobic forms and they quite likely evolved only after substantial levels of oxygen became available, due to oxygenic photosynthesis. If we further consider that the origin of anoxygenic photosynthesis and photosystem II occurred approx. 3.2–3.5 billion years ago (Blankenship, 2010), the origin of oxygenic photosynthesis at less than 3 billion years ago and the content of oxygen in the atmosphere increased slowly and gradually (over more than 2 billion years) to the actual level, it probably took billions of years before the anaerobic anoxygenic phototrophic bacteria lost their status as major and globally distributed players and were pushed back to remaining anoxic niches, which they still occupy today. During adaptation to oxic conditions, quite a number of anoxygenic phototrophic purple bacteria may have gained the ability to perform under both anoxic and oxic conditions by maintaining the strict regulation of biosynthesis of the photosynthetic apparatus and its repression by oxygen. An example of such bacteria is found in the *Rhodobacter* species performing anoxygenic photosynthesis under anaerobic conditions in the light and aerobic respiration under oxic conditions in the dark (Imhoff, 2005). Other phototrophic bacteria may have lost the ability to build up the photosynthetic apparatus in the absence of oxygen and in contrast require oxygen for formation of the photosynthetic apparatus. These latter represent the so called aerobic anoxygenic phototrophic bacteria with, e.g., *Erythrobacter* and *Roseobacter* species as examples (Shiba, 1991; Shimada, 1995).

The phylogenetic relationship of PufLM, as shown in **Figures 1, 2**, demonstrates that many of the aerobic phototrophic purple bacteria have a clear phylogenetic association to anaerobic members of the purple bacteria, though forming distinct phylogenetic lines (**Figures 2B,C**). This is true, e.g., for the aerobic phototrophic *Bradyrhizobium* species related to *Rhodopseudomonas palustris* and relatives and also for the aerobic *Limnohabitans planktonicus* related to the anaerobic *Rhodoferax fermentans*. This is of course also the case for the numerous Rhodobacteraceae that perform an aerobic type of phototrophic life and which are related to the anaerobic counterparts in the genera *Rhodobacter, Rhodobaca*, and *Rhodovulum*. Quite remarkable, members of these latter genera of anaerobic phototrophic bacteria are highly flexible in their metabolism and perform under strictly anoxic conditions by photosynthesis as well as under aerobic conditions by respiration, though the biosynthesis of the photosynthetic apparatus is repressed by oxygen and is formed under anoxic conditions in these bacteria (Imhoff, 2005; Imhoff, 2017). Other groups of aerobic phototrophic purple bacteria are more distantly related to known anaerobic counterparts. This is true for *Brevundimonas subvibrioides*, for all Sphingomonadales and to some extent also for *Methylobacterium* species, for *Fulvimarina pelagi* and *Caenispirillum salinarum* (**Figures 2B,C**).

*Gemmatimonas phototrophica* is an aerobic phototrophic bacterium and a representative of a new phylum, the Gemmatimonadetes. According to the 16S rRNA gene sequence, it is phylogenetically distant to Proteobacteria as well as to Chloroflexi (Zhang et al., 2003; Zeng et al., 2015). The PufLM sequences show distant relationship to some phototrophic Alphaproteobacteria (data not shown).

### Ecological Relevance of pufLM Sequences

The results of this study have strong implications on studies on the diversity and ecological relevance of phototrophic bacteria. Due to the specificity of *pufL* and *pufM* genes for anoxygenic phototrophic bacteria employing photosystem II and their presence in all of these bacteria, sequences of these genes are perfect tools to study the diversity of phototrophic purple bacteria in the environment (Tank et al., 2009, 2011; Thiel et al., 2010; Imhoff, 2017). This is of particular importance, because the 16S rRNA gene is not suited to analyze the environmental diversity of phototrophic bacteria, many of which show close relationships with chemotrophic relatives. As the phylogenetic identification of established species is prerequisite to identify these bacteria and their relatives in environmental metagenomes and to estimate the relative importance of individual species in nature, the primary use of sequences of type strains gives a reliable systematic background for species identification in environmental samples.

The data base of *pufLM* gene sequences of phototrophic purple bacteria (see **Supplementary Table S1**), in particular of type strains, is significantly enlarged by the present study and forms a solid reference for environmental studies that approach the diversity of phototrophic purple bacteria. A few case studies have already been made in which almost complete *pufLM* sequences successfully have been applied to diversity studies of purple sulfur bacteria (Gammaproteobacteria) in environmental samples and the determination of their species diversity (Tank et al., 2009, 2011; Thiel et al., 2010).

A comprehensive data base of complete *pufLM* sequences is of particular relevance, because a number of studies approaching the environmental diversity of phototrophic purple bacteria using the *pufL* and *pufM* genes, in particular the *pufM* gene (see Imhoff, 2016 for review) are hampered by one or more of at least three major pitfalls.

(i) First of all, due to primer selection, many of the studies have used a small part of the *pufM* gene only (often less than 300 nt), which turned out to be insufficient to achieve a clear species assignment.
(ii) Second, in most of these studies environmental sequences were insufficiently rooted with sequences of established species obtained from recognized types of these bacteria. Even though some of the studies have compared a large number of environmental sequences, too few reference sequences from known species have been available. Therefore, the nearest cultured neighbor of the environmental sequences could not be determined.
(iii) Third, sequences from known species often were not accurately identified by species name and strain number and in particular type strains have not been used. This results in unclear identification of the reference organisms and leaves doubt on whether the species name used is correct in a systematic/taxonomic sense.

Therefore, the comprehensive data base of type and reference strains from phototrophic purple bacteria with 197 *pufLM* gene sequences including 152 type strains and the demonstration of their phylogenetic relationship, is an excellent background for identification of species of phototrophic purple bacteria in environmental samples and a perfect tool to study their diversity and the adaptation of the species to changing environmental conditions (Tank et al., 2011).

## Conclusion

Although the advances in our knowledge on the diversity of phototrophic bacteria over the past decades are amazing, we may see only the “tip of the iceberg” of their diversity as it may exist or may have existed over the past billions of years. These advances are also reflected in changes of their systematic treatment. Prior to the establishment of 16S rRNA gene sequences for studies on diversity and systematics of the phototrophic purple bacteria, these were classified in the two families Rhodospirillaceae (purple non-sulfur bacteria) and Chromatiaceae (purple sulfur bacteria) (Pfennig and Trüper, 1974; Pfennig, 1977). The first consequences of phylogenetic relationships delineated from 16S rRNA gene sequences have been the recognition of the Ectothiorhodospiraceae as a separate family and a rearrangement of species within the Rhodospirillaceae (Imhoff, 1984, 2001, 2011; Imhoff et al., 1984). Woese (1987) already called the Proteobacteria “the phototrophic purple bacteria and their relatives.” Over the years that past since we gained increasing proof that the phototrophic purple bacteria indeed are deeply rooted within the Proteobacteria, with representatives in 10 recognized orders and at least 21 families. The high diversity of the phototrophic purple bacteria not only is seen in the diversity within the large groups of Chromatiales, Rhodobacterales, and Sphingomonadales, but it is most obviously visible by the large number of other groups and clusters, many of which are represented only by a few or even a single species at present.

The comprehensive view on PufLM sequence allows concluding on the existence of a common ancestor of phototrophic Proteobacteria having already a photosynthetic reaction center type-2 with PufL and PufM proteins as core components. The phototrophic Proteobacteria may share their ancestor with the Chloroflexales that quite likely separated very early in evolution of photosynthesis. A specific proof of the high diversity of bacteria with phototrophic capacities is given by the comprehensive phylogenetic analysis of highly conserved photosynthetic reaction center proteins PufL and PufM. As discussed and demonstrated in **Figure 1**, the PufLM phylogeny of the major groups including Chromatiaceae, Ectothiorhodaceae, Rhodobacterales, Burkholderiales, Sphingomonadaceae, and Erythrobacteraceae is in good agreement with the phylogeny of the 16S rRNA gene and does not give reason to consider lateral transfer of these genes. The heterogeneous appearance of distinct groups according to 16S rRNA gene sequences of Rhodospirillales and Rhizobiales in the phylogeny of PufLM is not well supported by bootstrap values and the data basis appears insufficient to make clear statements on possible lateral transfer of these genes within these orders. Though we do not want to exclude the lateral transfer of pufL and pufM genes, it is highly unlikely that this can explain their phylogenetic relations. A possible explanation for the uncertainty in the arrangement could be the early separation of individual groups of species and genera and the diversification of PufLM with different evolutionary rates. This could blur the phylogenetic traces.

As the pufLM genes are specifically associated with the property of anoxygenic photosynthesis, their sequences represent a most important and valuable tool for studies of environmental communities of phototrophic bacteria and enable to track the diversity of phototrophic purple bacteria in complex natural communities. For this purpose the presented data basis of sequences from reference and type strains makes it possible to correlate environmental sequences with well-established species.

## Author Contributions

Cultivation of bacterial cultures, DNA extraction and purification was performed by TR. Genomic sequencing and quality assurance by SK. Sequence assembly, retrieval from data bases, phylogenetic calculations and tree construction by SN. Sequence annotation, retrieval of sequences from annotated genomes and data bases, design of the study as well as writing of the manuscript was made by JI. All authors contributed to and approved the work for publication.

## Supplementary Material

TABLE S1 Sequences and deposition numbers of PufLM together with systematic information of the strains studied.

DATA SHEET S1 Alignment of PufLM sequences analyzed in this study.
